# Differentiation of human multipotent dermal fibroblasts into islet-like cell clusters

**DOI:** 10.1186/1471-2121-11-46

**Published:** 2010-06-25

**Authors:** Dan Bi, Fu Guo Chen, Wen Jie Zhang, Guang Dong Zhou, Lei Cui, Wei Liu, Yilin Cao

**Affiliations:** 1 Department of Plastic and Reconstructive Surgery, Shanghai 9thPeople's Hospital, Shanghai Jiao Tong University School of Medicine, Shanghai Key Laboratory of Tissue Engineering, National Tissue Engineering Center of China, Shanghai 200011, China

## Abstract

**Background:**

We have previously obtained a clonal population of cells from human foreskin that is able to differentiate into mesodermal, ectodermal and endodermal progenies. It is of great interest to know whether these cells could be further differentiated into functional insulin-producing cells.

**Results:**

Sixty-one single-cell-derived dermal fibroblast clones were established from human foreskin by limiting dilution culture. Of these, two clones could be differentiated into neuron-, adipocyte- or hepatocyte-like cells under certain culture conditions. In addition, those two clones were able to differentiate into islet-like clusters under pancreatic induction. Insulin, glucagon and somatostatin were detectable at the mRNA and protein levels after induction. Moreover, the islet-like clusters could release insulin in response to glucose in vitro.

**Conclusions:**

This is the first study to demonstrate that dermal fibroblasts can differentiate into insulin-producing cells without genetic manipulation. This may offer a safer cell source for future stem cell-based therapies.

## Background

Type 1 diabetes is an autoimmune disease that results in the destruction of insulin-producing beta cells of the pancreas [1]. Increasing evidence suggests that islet transplantation is a promising therapy for type 1 diabetes. However, the limited supply of donors for pancreatic islets severely limits this approach. The use of stem cells to produce a new population of functional beta cells may offer an alternative approach. Embryonic and adult stem cells, including mesenchymal stem cells, hepatic oval cells, adult pancreatic stem cells, and pancreatic-ducts stem cells, are able to differentiate into insulin-secreting cells in vitro and correct hyperglycemia in diabetic animal models [2-8].

Recent studies have demonstrated that dermal fibroblasts can be reprogrammed into embryonic stem cell-like cells called induced pluripotent stem (iPS) cells by introducing several stem cell-associated genes [9,10]. Tateishi et al. showed that dermal fibroblast-derived iPS cells could also generate insulin secreting islet-like cells [11], suggesting that iPS cells might be a potential cell source for therapy. However, the genetic manipulation involved could be problematic for future clinical applications.

In our previous study, we isolated, expanded and characterized one clonal population from dermal fibroblasts, which is able to differentiate into mesodermal, ectodermal and endodermal progenies in vitro [12]. It is of great interest to know whether these cells can be further differentiated into functional insulin-producing cells. Thus, the aim of this study was to establish clones from the human dermis with greater multipotency, and to investigate their potential to differentiate into pancreatic cells.

## Results

### Differentiation potential of single-cell derived dermal fibroblasts

**Figure 1:**
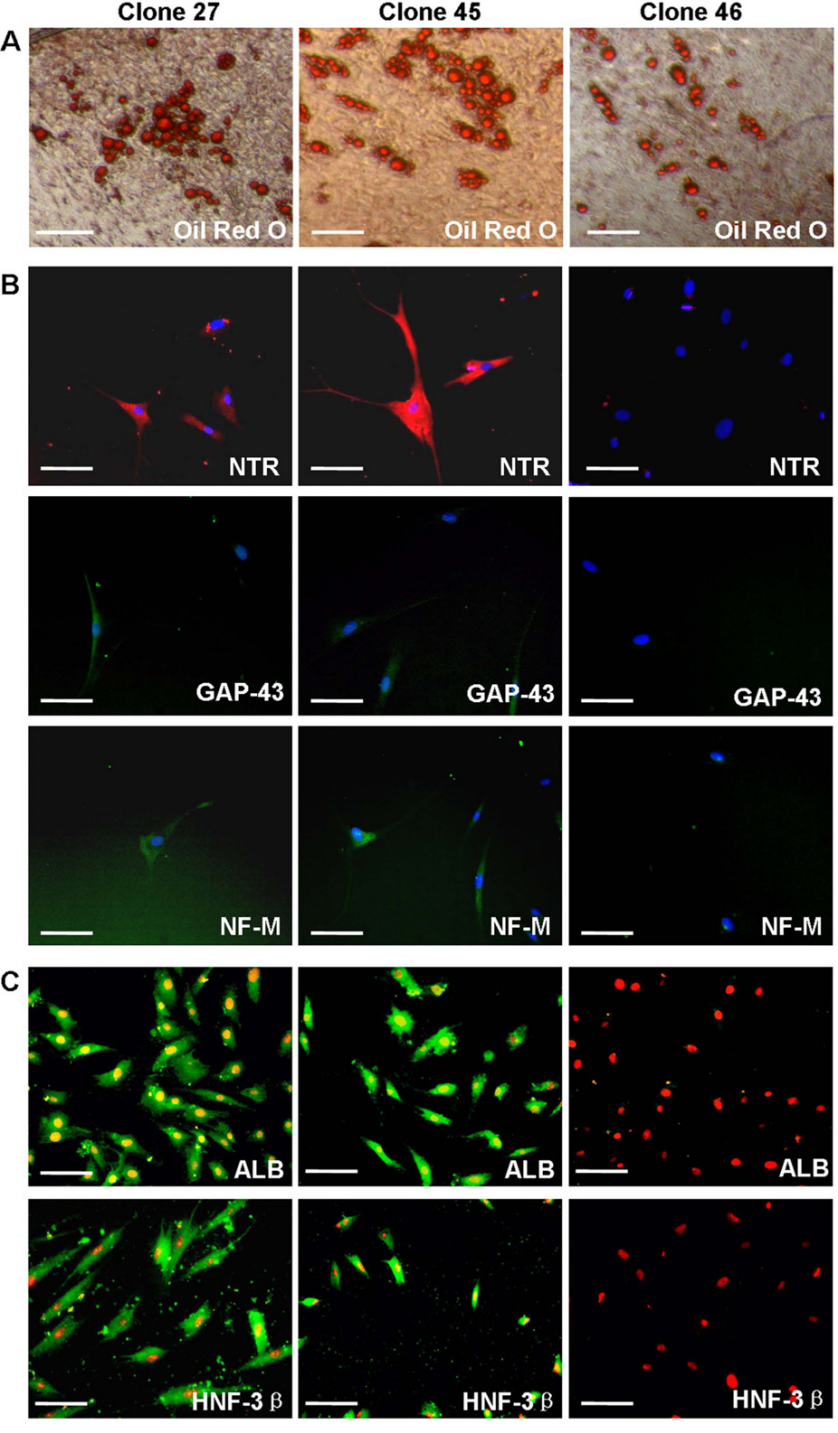
**Differentiation potential of single-cell-derived dermal fibroblast clones**. Cell were expanded for over 25 doublings and were then induced in differentiation media for the time intervals described in the Materials and Methods. Adipogenesis was evaluated by Oil Red O staining (A). Neurogenesis (B) was evaluated by anti-neurotensin receptor 3 (NTR, red color), anti-growth associated protein-43 (GAP-43, green color), and anti-neurofilament (NF-M, green color) staining. Hepatogenesis (C) was demonstrated by anti-albumin (ALB, green color) and anti-hepatic nuclear factor 3 beta (HNF-3β, green color) staining. Scale bars, 100 μm.

In 70 of the 96-well plates (five plates/donor) seeded, 150 wells contained a single-cell-derived clone, but only 61 clones could survive and be continuously expanded. The established clones exhibited a spindle-shaped morphology without obvious differences between each clone (data not shown). After 23-30 cell-doublings, the cells were cultured in adipogenic, neurogenic and hepatogenic media to determine their differentiation potential. As expected, 34% (21/62) of the clones exhibited adipogenic differentiation potential and were positive for Oil-Red O staining after induction for 3 weeks (Figure 1A). Of these, only two clones (Clones 27 and 45) showed neurogenic and hepatogenic potential, as demonstrated by anti-NTR-3, anti-GAP-43, anti-NF-M (Figure 1B), anti-ALB, and anti-HNF-3β staining (Figure 1C). Therefore, these two clones were termed multipotent dermal fibroblasts (MDFs). Interestingly, none of the clones possessed neurogenic or hepatogenic potential alone. These findings were consistent with those observed in our previous study [12].

### In vitro differentiation of MDFs into islet-like clusters

**Figure 2:**
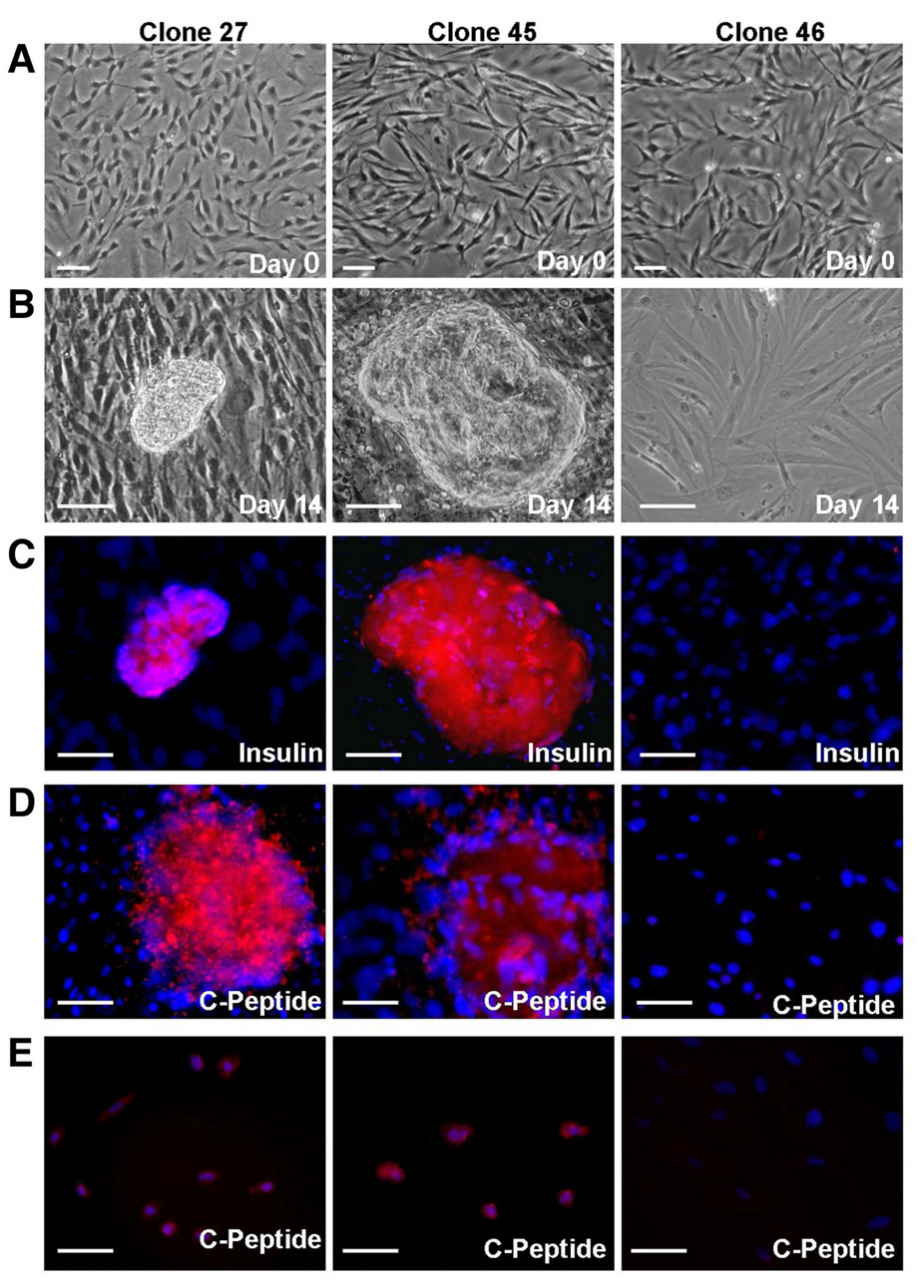
**Differentiation of MDFs into islet-like clusters**. Single-cell derived MDF clones (Clone 27 and 45) exhibited a spindle-shaped morphology in culture (A) and formed cell clusters after 14 days of pancreatic induction (B). The cell clusters were positive for anti-insulin (C, red color) and anti-C-peptide (D, red color) antibody staining. Single cells from cell clusters were also positive for anti-C-peptide (E, red color) antibody staining. The nucleus is stained with DAPI (blue). Cell clusters and positive antibody staining were not observed in Clone 46, with or without induction. Scale bars, 100 μm.

The existence of multipotent cells in cultured dermal fibroblasts prompted us to check whether these cells could be further differentiated into insulin producing cells. Clones 27 and 45 (about 25 cell-doublings) were treated with factors known to promote pancreatic differentiation. Clone 46, which only possesses adipogenic potential, was used as a control. After 14 days of induction, 20-30 (26 ± 1.7) of spherical cell clusters were observed in each well of the six-well plate in Clones 27 and 45. However, cell clusters were not observed in Clone 46, or in the non-induced group (Figure 2A and 2B). Immunofluorescent staining showed that the cell clusters were positive for anti-insulin antibody staining (Figure 2C). No staining was observed in the isotypic control group (data not shown). It has been reported that immunoreactivity of insulin in the cell clusters might due to the uptake of insulin from culture media [13]. Therefore, pancreatic differentiation was further confirmed by anti-C-peptide antibody staining (Figure 2D). Positive staining was observed in Clone 27 and 45. In addition, to rule out the non-specific staining, cell clusters were picked out, dissociated by typsin, and single cells were seeded on cover glasses for 2 hours. Immunofluoresent staining for anti-C-peptide was then repeated. Again, positive staining was observed in Clone 27 and 45, but not in Clone 46 (Figure 2E).

**Figure 3:**
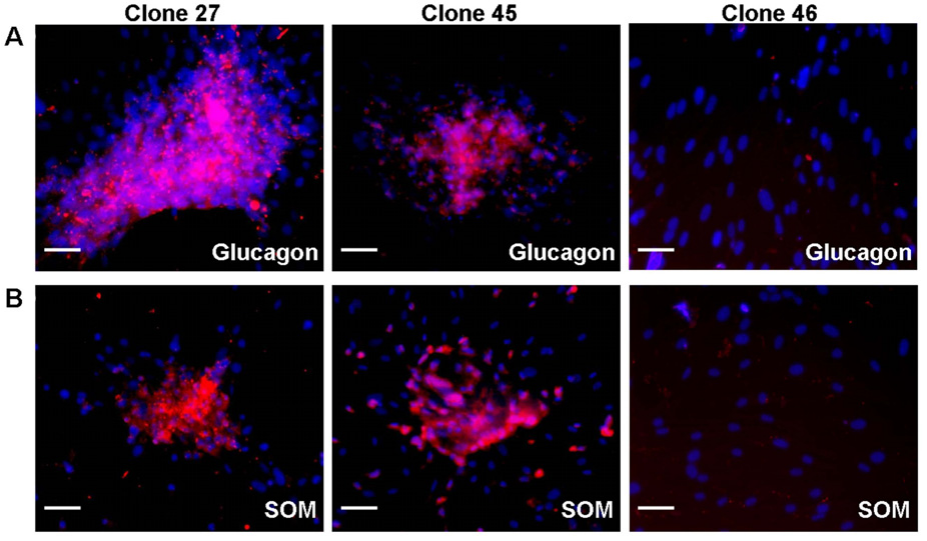
**Expression of glucagon and somatostatin (SOM) in MDFs derived islet-like clusters**. Islet-like clusters derived from Clone 27 and 45 after 14 days of pancreatic induction were positive for anti-Glucagon (A) and anti-SOM (B) staining (red), but negative in Clone 46. Nuclei were stained with DAPI (blue). Scale bars, 100 μm.

To characterize the pancreatic differentiation in more detail, immunofluorescent staining of other pancreatic endocrine hormones was performed, including glucagon, which is synthesized and secreted from alpha cells of the islets, and somatostatin, which is secreted from delta cells of the islets. Interestingly, the cell clusters in the induced Clones 27 and 45 were positive for anti-glucagon and anti-somatostatin staining, whereas Clone 46 was not (Figure 3A, B).

**Figure 4:**
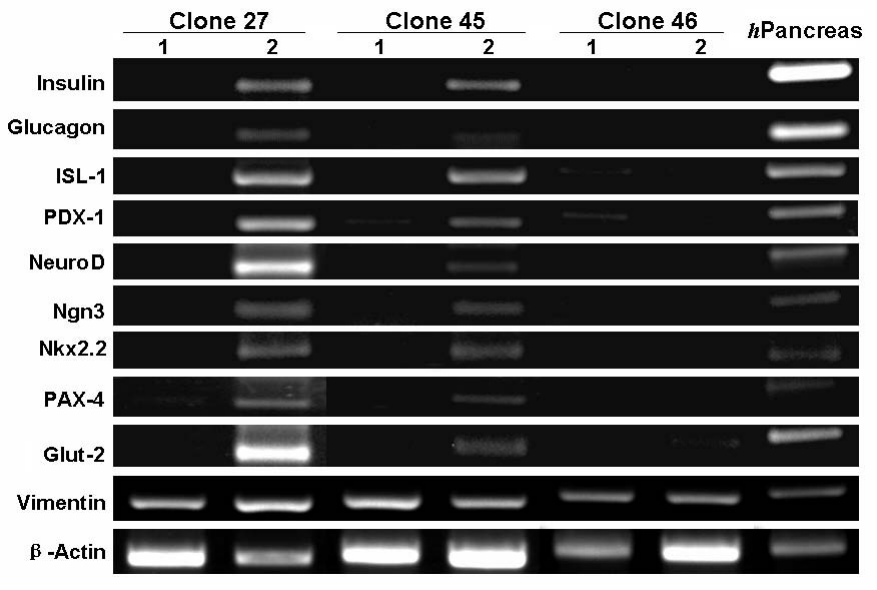
**Pancreatic gene expression in MDFs**. Cells before (1) and after 14 days of pancreatic induction (2) were harvested and analyzed by RT-PCR. Human adult pancreatic tissue was used as a positive control. Only for Ngn3, RNA isolated from fetal pancreas was used as control.

The gene expression profile analyzed by RT-PCR further confirmed the pancreatic differentiation of MDFs. As shown in Figure 4, pancreatic developmental transcription factors, including PDX-1, ISL-1, NeuroD, Nkx 2.2, Ngn3 and PAX-4, were not detectable in the non-induced cells. By contrast, these factors were upregulated in induced MDFs (Clones 27 and 45). The islet-specific hormones insulin and glucagon were also upregulated in these cells after pancreatic induction, together with a glucose transporter gene (Glut-2). By contrast, there was no expression of pancreatic genes in Clone 46, with or without induction. Vimentin, the mesoderm marker, was expressed in all of the cells tested here. All of these data support the concept that MDFs can be differentiated into islet-like cells under pancreatic induction.

### Release of insulin in response to glucose stimulation

**Figure 5:**
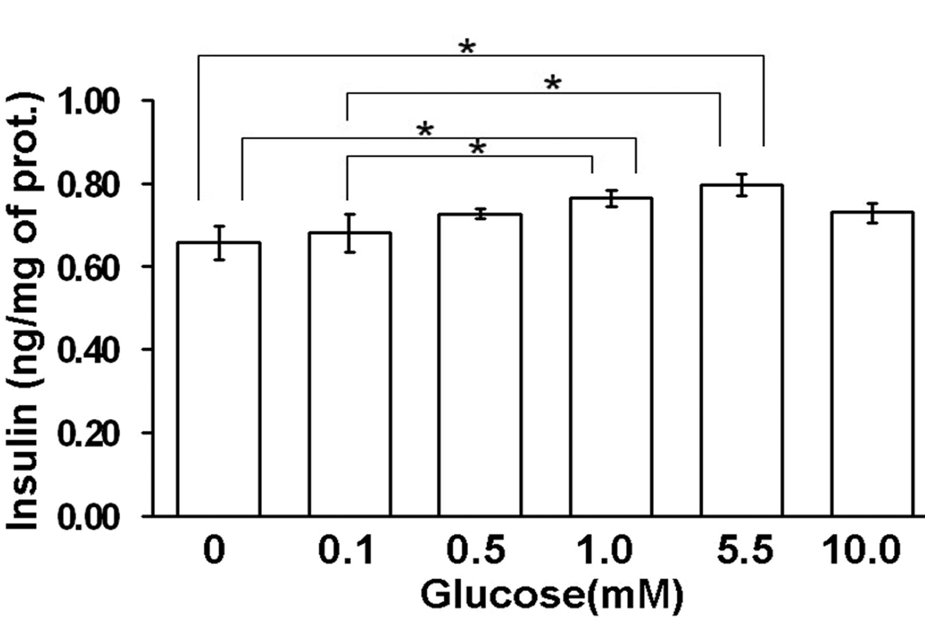
**Islet-like cell clusters release insulin in response to glucose in vitro**. After pancreatic induction, the amount of insulin in the culture media was measured by ELISA after the cells (clones 27 and 45) were exposed to different concentrations of glucose. One-way ANOVA showed significant differences between groups (p < 0.05). The asterisks denote a significant difference between two groups.

To determine whether the differentiated MDFs (Clones 27 and 45) were responsive to glucose challenge, insulin release after exposure to high glucose was measured using human insulin ELISA kit. Clone 27 and 45 were measured separately and each clone was repeated three times. Similar pattern of glucose response were achieved in both clones. Therefore, data from those two clones were summarized in Figure 5. The insulin secretion was gradually increased with the increase in glucose concentration from 0 mM to 5.5 mM in differentiated MDFs. However, secretion did not increase further at a higher glucose concentration (10 mM). One-way ANOVA showed significant differences between groups (p < 0.05). Comparing with non-stimulated group (0 mM), significant differences (P < 0.05) were observed in the groups with glucose stimulation at 1 mM or 5.5 mM, but not in the other groups (0.1 mM, 0.5 mM, 10 mM). In addition, significant differences were also observed in those two groups (1 mM, 5.5 mM) comparing with the one stimulated with glucose at 0.1 mM. These data indicate that islet-like cell clusters derived from MDFs could secrete insulin in response to glucose stimulation at a very limited level.

## Discussion

Numerous studies have demonstrated the presence of stem cells in the adult dermis [14,15]. Our previous study revealed that dermal fibroblasts are a heterogeneous population that contains cells with variable differentiation potential [12]. In the present study, we confirmed that a small population of cells from human foreskin is multipotent. These cells could be differentiated into neuron-, adipocyte- and hepatocyte-like cells in the presence of certain factors. More importantly, these cells could differentiate into islet-like cells capable of expressing insulin, glucagon and somatostatin after pancreatic induction. In addition, the islet-like clusters could release insulin in response to glucose in vitro.

Pancreatic differentiation of MDFs has been demonstrated by the expression of pancreatic genes at the mRNA (Figure 4) level. Although, immunoreactivity of insulin was observed in the cell clusters after induction (Figure 2C), it can not rule out the possibility that the insulin is trapped in clusters, since the media contains FBS/insulin [13]. By anti-C-peptide immunostaining, we confirmed that induced cells do express insulin (Figure 2D, E). Unfortunately, the functional assay showed that the islet-like cells released insulin in response to glucose stimulation with limited ability (Figure 5). The low insulin secretory capacity may be due to the loss of stem cell properties during MDF expansion. We have found that cells after 30 cell-doublings completely lose their multipotency (data not shown). Thus, the cells investigated in this study (approximately 25 cell-doublings) were close to their capacity. Optimal cell expansion conditions are worth to be investigated to improve the pancreatic differentiation ability. Meanwhile, the in vivo function of insulin-producing cells is under investigation in diabetic animals.

Vimentin is the marker of mensenchymal cells. The MDFs express vimentin even after pancreatic induction (Figure 4). Although there have been reports of epithelial-to-mesenchymal transition (EMT) of pancreatic islet cells and differentiation of such cells back to insulin-producing cells [16-18], it is hard to believe that skin epithelial cells undergo EMT to differentiate into pancreatic cells. Guo et al. reported that skin-derived precursors (SKPs) from mouse, which express nestin but not vimentin, could also be differentiated into insulin producing cells[19], indicating that EMT is not likely the mechanisms for differentiation of skin cells to pancreatic cells. As we discussed previously, the relationship between SKPs and the multipotent dermal fibroblasts in our study is still unclear [12]. Nevertheless, it supports our findings that skin contains multipotent cells that could be differentiated into islet-like cells.

The finding that MDFs could give rise to cells of three germ layers is surprising. Interestingly, similar types of multipotent cells have been isolated from other adult tissues, such as spermatogonial stem cells in the adult testis [20] and very small embryonic-like (VSEL) stem cells in the bone marrow [21]. Kucia et al. speculated that the multipotent stem cells in adult tissues might be derived from early embryonic cells that reside in different tissues during developmental process and are maintained to the adult stage [21]. It could be speculated that those MDFs might be derived from residual undifferentiated cells in the dermis. To further localize these cells in the dermis and to enrich them from the pooled population, the characteristics unique to these cells are under investigation.

Recent studies have demonstrated that the iPS cells derived from reprogramming of dermal fibroblasts are multipotent and could generate insulin secreting islet-like cells [11]. The relationship between MDFs and iPS cells should also be investigated. Kim et al. demonstrated that the induction of iPS from stem cells seems to be easier than that from differentiated cells [22]. One possibility is that the reprogramming process might mainly occur in MDFs rather than in the differentiated dermal fibroblasts. Thus, only MDFs could selectively expand in culture. Moreover, the number of reprogramming factors could be reduced when using MDFs as a target. Further studies are needed to answer these questions.

## Conclusions

In summary, this is the first study to demonstrate that multipotent dermal fibroblasts could be differentiated into islet-like cells without genetic manipulation. This approach might offer a safer cell source for future stem cell-based therapies.

## Methods

### Derivation of single-cell derived clones from dermis

Single-cell derived clones were established according to the method described by Chen et al. [12]. Briefly, fresh human foreskin specimens were obtained from 14 donors aged from 6 to 12 years who underwent a routine circumcision procedure at Shanghai Children's Hospital with informed consent. The study was performed with approval from the local ethics committee (Shanghai Jiao Tong University School of Medicine, China). The single-cell suspension was achieved after enzyme digestion and filtration. The cells were then suspended in high-glucose DMEM (DMEM-HG; Invitrogen Corporation, Carlsbad, CA, USA), supplemented with 10% FBS (HyClone, Logan, UT), 300 μg/m1 L-glutamine, 50 μg/ml vitamin C, 100 U/ml penicillin and 100 μg/ml streptomycin (all from Sigma, St Louis, MO, USA). The cells were then seeded in 96-well tissue culture plates (Falcon, Franklin Lakes, NT) at a concentration of 2 cells/well (200 μl/well, five plates/donor). After 24 h, the plates were scored under the microscope. Wells containing only one cell were marked for further analysis. The cells were kept in the original culture medium at 37°C with 5% CO_2_. After 2-3 weeks, the single-cell-derived clones were harvested and transferred to 24-well and six-well tissue culture plates (Falcon) for expansion. In total, 61 clones were established, and their differentiation potential was analyzed.

### Multilineage differentiation of single-cell derived clones

Single-cell-derived clones were analyzed for their capacity to differentiate into adipocytes, neuron and hepatocytes. Each experiment was repeated at least three times for cells from each clone.

### Adipogenic differentiation

Adipogenic differentiation was performed as previously described [12]. Briefly, cells were seeded at 1 × 10^3 ^cells/cm^2 ^in adipogenic medium for 3 weeks. Cells cultured in the regular culture medium were used as a control. The medium was changed every 3 days. Cells were stained with Oil Red O reagent (Sigma) for 5 minutes after induction.

### Neurogenic differentiation

Neurogenic differentiation was performed as previously described [23]. Briefly, cells were seeded at 1 × 10^3 ^cells/cm^2 ^on fibronectin-coated dishes, incubated with sequential changes in neurogenic medium for up to 10 days. Cells maintained in regular culture medium were used as a control.

### Hepatogenic differentiation

Hepatogenic differentiation was performed as described by Schwartz et al. [24]. Cells were seeded at 4 × 10^3 ^cells/cm^2 ^on fibronectin-coated dishes, pre-induced for 10 h followed by culturing with hepatogenic medium for up to 3 weeks. The medium was changed every 2 days. Cells maintained within the regular culture medium were used as a control.

### Pancreatic differentiation of multipotent dermal fibroblasts in vitro

To induce the multipotent dermal fibroblasts (MDFs) to undergo pancreatic endocrine cell differentiation, the cells were treated by sequential specification, commitment and differentiation with factors known to promote the expression of a beta cell-like phenotype, as previously described [2,25-27]. Briefly, cells were plated at a density of 10,000 cells/cm^2 ^in the presence of DMEM-HG, 20% FBS for 24 hours. On the next day, medium containing DMEM-HG, 20% FBS and 10 ng/ml b-FGF (#233-FB-025; R&D Systems, Minneapolis, MN) was added for another 24 hours. Endodermal specification was then induced by exposure to medium containing DMEM-HG, 1% DMSO (#D4540; Sigma), 100 mM butylated hydroxyanisole (BHA, #B1253; Sigma), and 10 nM exendin-4 (#E7144; Sigma) for 24 hours. Cells were washed three times, and endodermal commitment was induced by exposure to medium containing RPMI 1640 (#12633-012; Invitrogen), 11.1 mM glucose, 10% FBS, 1.0 mM sodium pyruvate, 10 mM Hepes (#H3375; Sigma),20 ng/ml b-FGF and 20 ng/ml EGF (#236-EG-200; R&D Systems), and 10 nM exendin-4 for 4 days. Islet-like differentiation was subsequently induced by exposing the cells to medium composed of RPMI 1640, 2.5 mM glucose, 10 mM Hepes, 10 mM nicotinamide (#N0636; Sigma), 100 pM HGF (#H1404; R&D Systems), 10 nM exendin-4, and 2.0 nM activin-A (#A4941; Sigma) for 5-7 days.

### Immunofluorescent staining

To stain intracellular proteins, the cells were fixed in 4% cold paraformaldehyde (Sigma) in phosphate-buffered saline (PBS) for 15 minutes on chamber slides, and permeabilized with 0.25% Triton X-100 (Sigma) in PBS for 10 minutes. The blocking and diluent solutions comprised PBS, 1% BSA and 1% serum (Sigma) from the species in which the secondary antibody was raised. The fixed cells were blocked for 30 minutes, incubated sequentially with the primary antibodies diluted in PBS at 4°C overnight, followed by incubation with secondary antibodies. Cells exposed to isotypic antibodies were used as controls. Nuclei were counterstained by DAPI (4', 6'-diamidino-2-phenylindole, Invitrogen). The primary antibodies, including anti-neurotensin receptor 3 (NTR-3, 1:100), anti-growth associated protein-43 (GAP-43, 1:100), anti-neurofilament (NF-M, 1:100), anti-hepatic nuclear factor 3 beta (HNF-3β, 1:100), anti-albumin (ALB, 1:100), anti-insulin (1:100), anti-C-peptide (1:100), anti-glucagon (1:100) and anti-somatostatin (SOM, 1:100) were purchased from Santa Cruz Biotechnology. Secondary antibodies, including anti-goat IgG (H+L), anti-rabbit IgG (H+L), anti-mouse IgG (H+L) labeled with Alexa Fluor 488 or Alexa Fluor 546 (1:1000 Molecular Probes, Invitrogen), were used according to the manufacturer's recommendations.

### RT-PCR analysis

Total RNA was extracted with TRIzol (Invitrogen) and reverse-transcribed into cDNA with an RT-PCR kit (TaKaRa, Shiga, Japan). The primer sequences (all from Sangon Technology Co., Shanghai, China), reaction conditions and the sizes of each product are listed in Table 1. The amplified products were separated on 1.5% agarose gels and visualized with ethidium bromide. Human adult pancreatic tissue was used as a positive control. Only for Ngn3, which is not expressed in adult pancreas, RNA isolated from fetal pancreas was used as control.

**Table 1:** Primers used in RT-PCR analysis

Gene	Primer sequence	Number of cycles	Annealing temperature (°C)	Product size(bp)
Insulin	Sense: 5'-GCCTTTGTGAACCAACACCTG -3' Antisense: 5'-GTTGCAGTAGTTCTCCAGCTG -3'	35	67	261
Glucagon	Sense: 5'-ATCTGGACTCCAGGCGTGCC -3' Antisense: 5'-AGCAATGAATTCCTTGGCAG -3'	35	65	170
ISL-1	Sense: 5'-CAACAAACAAAACGCAAAAC -3' Antisense: 5'-AAGTCAAACACAATCCCGA -3'	35	62	542
PDX-1	Sense: 5'-GTCCTGGAGGAGCCCAAC -3' Antisense: 5'-GCAGTCCTGCTCAGGCTC -3'	35	65	360
NeuroD	Sense: 5'-GAAAGCCCTCTGACTGAT -3' Antisense: 5'-AAACTGGCGTGCCTCTAA -3'	35	58	314
Ngn3	Sense: 5'-GTAGAAAGGATGACGCCTCAACC-3' Antisense: 5'-TCAGTGCCAACTCGCTCT TAGG-3'	35	62	241
Nkx2.2	Sense: 5'-TTCTACGACAGCAGCGACAACC-3' Antisense: 5'-CGTCACCTCCATACCTTTCTG-3'	35	62	393
Pax-4	Sense: 5'-GGGGCTCTTTGTGAATGG-3' Antisense: 5'-CCGCAGGACTCGGTTGAT-3'	35	58	349
Glut-2	Sense: 5'-CCGCTGAGAAGATTAGAC-3' Antisense: 5'-CTGGATACAGACAGGGAC-3'	35	58	492
Vimentin	Sense: 5'-CCAGGCAAAGCAGGAGTC-3' Antisense: 5'-GGGTATCAACCAGAGGGAGT-3'	35	62	380
β-actin	Sense: 5'-CCAAGGCCAACCGCGAGAAGATGAC-3' Antisense: 5'-AGGGTACATGGTGGTGCCGCCAGAC-3'	35	55-67	592

Abbreviations: ISL-1, islet-1 transcription factor; PDX-1, pancreatic duodenal homeobox-1; NeuroD, neurogenic differentiation; Ngn3, neurogenin 3; Nkx2.2, NK-2 family homeodomain transcription factor; Pax-4, paired box gene 4; Glut-2, glucose transporter 2.

### Glucose-regulated insulin secretion

To determine whether cells could respond to glucose in vitro, the differentiated cells were pre-incubated for 4 hours at 37°C in Krebs-Ringer bicarbonate Hepes (KRBH) buffer containing 118 mM sodium chloride, 4.7 mM potassium chloride, 1.1 mM potassium dihydrogen phosphate, 25 mM sodium hydrogen carbonate, 3.4 mM calcium chloride, 2.5 mM magnesium sulfate, 10 mM Hepes and 2 mg/ml bovine serum albumin (BSA). For high-glucose-induced insulin release, cells were incubated in KRBH buffer supplemented with different concentrations of glucose (0-10 mM) together with 10 μM tolbutamide (Sigma) for 2 hours at 37°C. The concentration of insulin secreted into the culture media was measured using an ultra-sensitive human insulin enzyme-linked immunosorbent assay (ELISA) kit (Diagnostic Systems Laboratories, Webster, TX, USA). Cells were collected and total protein was determined using the DC protein assay system (Bio-Rad, Hercules, CA, USA). The data are presented as secreted insulin/total protein (ng/mg). Clone 27 and 45 were measured separately and each clone was repeated three times.

### Statistical analysis

Each experiment was repeated at least three times. All data are presented as means ± standard deviation. The data of insulin release after glucose stimulation were subjected to one-way analysis of variance (ANOVA) using the SPSS version 13.0, and the Student Newman-Keuls test was used for multiple comparisons. *P *values less than 0.05 are interpreted as significant.

## Authors' contributions

DB established single cell clones, carried out the cell differentiation studies (fluorescence imaging, RT-PCR) and insulin release study, and drafted the manuscript. FGC helped in establishment of clonal cells. GDZ, LC and WL helped in the design of the study. WJZ and YLC conceived the study, and participated in its design and coordination and helped to draft the manuscript. All authors have read and approved the final manuscript.
